# Pulsed Focused Ultrasound Reduces Hippocampal Volume Loss and Improves Behavioral Performance in the Kainic Acid Rat Model of Epilepsy

**DOI:** 10.1007/s13311-023-01363-7

**Published:** 2023-03-14

**Authors:** Po-Chun Chu, Chen-Syuan Huang, Shan-Zhi Ing, Hsiang-Yu Yu, Robert S. Fisher, Hao-Li Liu

**Affiliations:** 1 Department of Electrical Engineering, National Taiwan University, Taipei, Taiwan; 2 School of Veterinary Medicine, National Taiwan University, Taipei, Taiwan; 3 Department of Neurology, Taipei Veteran General Hospital, Taipei, Taiwan; 4 School of Medicine, National Yang Ming Chiao Tung University, Taipei, Taiwan; 5 Department of Neurology and Neurological Sciences, Stanford University School of Medicine, Palo Alto, CA USA

**Keywords:** Focused ultrasound, Neuromodulation, Temporal lobe epilepsy, Kainic acid model

## Abstract

Focused ultrasound (FUS) has the potential to modulate regional brain excitability and possibly aid seizure control; however, effects on behavior of FUS used as a seizure therapy are unknown. This study explores behavioral effects and hippocampal restoration induced by pulsed FUS in a kainic acid (KA) animal model of temporal lobe epilepsy. Twenty-nine male Sprague–Dawley rats were observed for 20 weeks with anatomical magnetic resonance imaging (MRI) and behavioral performance evaluations, comprising measures of anxiety, limb usage, sociability, and memory. FUS targeted to the right hippocampus was given 9 and 14 weeks after KA was delivered to the right amygdala. Ultrasound pulsations were delivered with the acoustic settings of 0.25 of mechanical index, 0.5 W/cm^2^ of intensity spatial peak temporal average (I_SPTA_), 100 Hz of pulse repetition frequency, and 30% of duty cycle, during three consecutive pulse trains of 10 min separated by 5-min rests. Controls included normal animals with sham injections and KA-exposed animals without FUS exposure. Longitudinal MRI observations showed that FUS substantially protected hippocampal and striatal structures from KA-induced atrophy. KA alone increased anxiety, impaired contralateral limb usage, and reduced sociability and learning. Two courses of FUS sonications partially ameliorated these impairments by enhancing exploring and learning, balancing limb usage, and increasing social interaction. The histology results indicated that two sonications enhanced neuroprotection effect and decreased the inflammation markers induced by KA. This study supports existence of both neuroprotective and beneficial behavioral effects from low-intensity pulsed ultrasound in the KA animal model of epilepsy.

**Supplementary Information:**

The online version contains supplementary material available at 10.1007/s13311-023-01363-7.

## Introduction

Epilepsy is diagnosed in approximately 50 per 100,000 individuals per year [1]. The conceptional definition of epilepsy [2] includes “the neurobiologic, cognitive, psychological, and social consequences of this condition.” Temporal lobe epilepsy (TLE) is the most common form of epilepsy in adults, characterized by focal aware (previously called simple partial) seizures or focal impaired awareness (previously called complex partial [3]) seizures, and seizures that may generalize secondarily. Accompanying features include ictal and interictal electroencephalographic (EEG) abnormalities, commonly coupled with behavioral dysfunction and hippocampal sclerosis [4–6]. Cognitive impairment, particularly affecting memory, is a major concern in TLE. Depression, stress, and anxiety are comorbid disorders that can exacerbate declines in cognition and overall quality of life. Seizures, medications, underlying brain injuries, and circuit reorganization all may contribute to cognitive and emotional comorbidities of epilepsy.

Effects of seizures on behavior and potential amelioration by focused ultrasound (FUS) can be studied in animal models of epilepsy. A commonly employed model originally described by Ben-Ari et al. [7] is generated by administration of kainic acid (KA), a cyclic analog of l-glutamate and an agonist of ionotropic KA receptors. Intracerebral administration of KA to the amygdala or hippocampus induces behavioral seizures and neuropathological lesions that are similar to those occurring in patients with TLE [6]. Resulting neuronal degeneration in the CA3 region of the dorsal hippocampus and ventral striatum involve networks important for memory processes and related behaviors [8]. Therefore, exploration of animal models can lead to a better understanding of the basic mechanisms involved in TLE-associated cognitive deficits.

FUS is a novel technology that can transcranially and non-invasively target deep brain tissue. Low-intensity FUS with neuromodulatory effects, below levels producing lesions, has been reported to reduce seizures and brain injury in animal models [9–12]. FUS sonication suppressed the number of epileptiform EEG spike bursts [9] and interfered with the PI3K-Akt-mTOR pathway [13]. The neuroprotective/behavioral potential of transcranial ultrasound stimulation in chronic epilepsy was suggested by laboratory studies showing that ultrasound stimulation delivered around the time of seizure onset can inhibit epileptiform EEG activity, concomitant with improvements in sociability, markers of depression [12], and inhibition of the expression of inflammatory factors and apoptosis-related proteins [14] in a small animal epilepsy model. FUS also improved electrophysiological measures and behavioral outcomes in a non-human primate model of epilepsy [15]. We previously showed that low-intensity pulsed FUS (0.25-MI, 30% duty cycle, 100 Hz pulse repetition frequency (PRF), 0.5 W/cm^2^ spatial-peak temporal average intensity (I_SPTA_)) can have long-term substantial effect to suppress EEG epileptiform spikes, bursts, and EEG seizures in a KA-induced epilepsy model for 7 weeks without tissue injury [16]. This preliminary study indicated that sonication reduced inflammatory glial fibrillary astrocytic protein (GFAP) markers and restored hippocampal morphology from histological examinations. Confirmation of the neuroprotective and long-term behavioral effects of FUS stimulation would be useful prior to exploring FUS as a clinical therapy for epilepsy [17].

This study aims to explore the neuroprotective efficacy of pulsed FUS neuromodulation in the KA model studied with behavioral analysis over a long term (4 months) [18]. In addition to providing evidence of morphological and histological restorations induced by pulsed FUS neuromodulation in KA model, longitudinal magnetic resonance imaging (MRI) examinations were conducted to evaluate hippocampal and striatal structural changes induced by KA. IBA-1 and tumor necrosis factor alpha (TNF-α) of immunohistochemistry (IHC) staining was performed to additionally evaluate the neuroprotective effects of FUS treatments.

## Methods

### Animals

All animal experiments were approved by the Institutional Animal Care and Use Committee of National Taiwan University, Taiwan (IACUC No. NTU-109-EL-00101). Animals were housed with a 12-h light/dark cycle and ad libitum access to food and water. Thirty male Sprague–Dawley rats (290–330 g, BioLASCO Co., Ltd., Taiwan) were used. Among them, 15 received KA injections and 14 survived; 15 received non-KA sham injection. There were 10/10/12 MRI scans for animals at three MR scanning time points, respectively: non-KA scans = 3/3/3, KA-only scans = 3/3/5, and KA + FUS scans = 4/4/4 (animals were randomly picked up from those with MR-allowable animal size). On the other hand, twenty-two rats were evaluated experimentally with behavioral tasks. Of these, 6 KA-injected rats were treated with FUS, and 16 rats (7 KA-injected rats and 9 non-KA-injected rats) were used as non-FUS controls.

### Kainic Acid Animal Model

The kainic acid model of temporal lobe epilepsy presents with neuropathological and electroencephalographic features of temporal lobe epilepsy, with a chronic period of recurrent seizures [6]. The KA model was developed based on previous literature [19], because of its similarity to temporal lobe seizures in humans. In brief, all animals were anesthetized with 2% isoflurane mixed with oxygen at 6 L/min. The skull was exposed and opened with a dental drill. KA 750 ng was injected into the right amygdala (AP: − 2.3 mm, ML: + 4.5 mm, DV: − 8.2 mm from bregma [20]) through a glass capillary tubing (Polymicro Technologies, Arizona, USA) under 20 ng/s of flow rate to induce excitotoxic damage in hippocampus. After injection, the glass cannula remained in place for 5 min to prevent leakage along the injection tract. Control animals without kainic acid injections were designated as “non-KA” animals. The wound was closed with sutures and tissue adhesives. After KA intra-amygdala injection, convulsive status epilepticus developed and lasted for at least 4 h in all animals. Thirteen of the fourteen Sprague–Dawley rats (92%) survived the kainic acid–induced status epilepticus. In our previous study, all KA-injected animals exhibited increasing EEG spikes and electrographic seizures over time (Supplementary S1) [16]. Our historical data showed that the animals receiving KA had bursts (defined as at least 3 closely spaced spikes) per 8 h, increasing from an average of 859 at week 2 to 1255 at week 7 along with a mean of 4 electrographic seizures per week, which was significantly more than those of the non-KA group [16].

### FUS Setup and Parameter Design

**Fig. 1:**
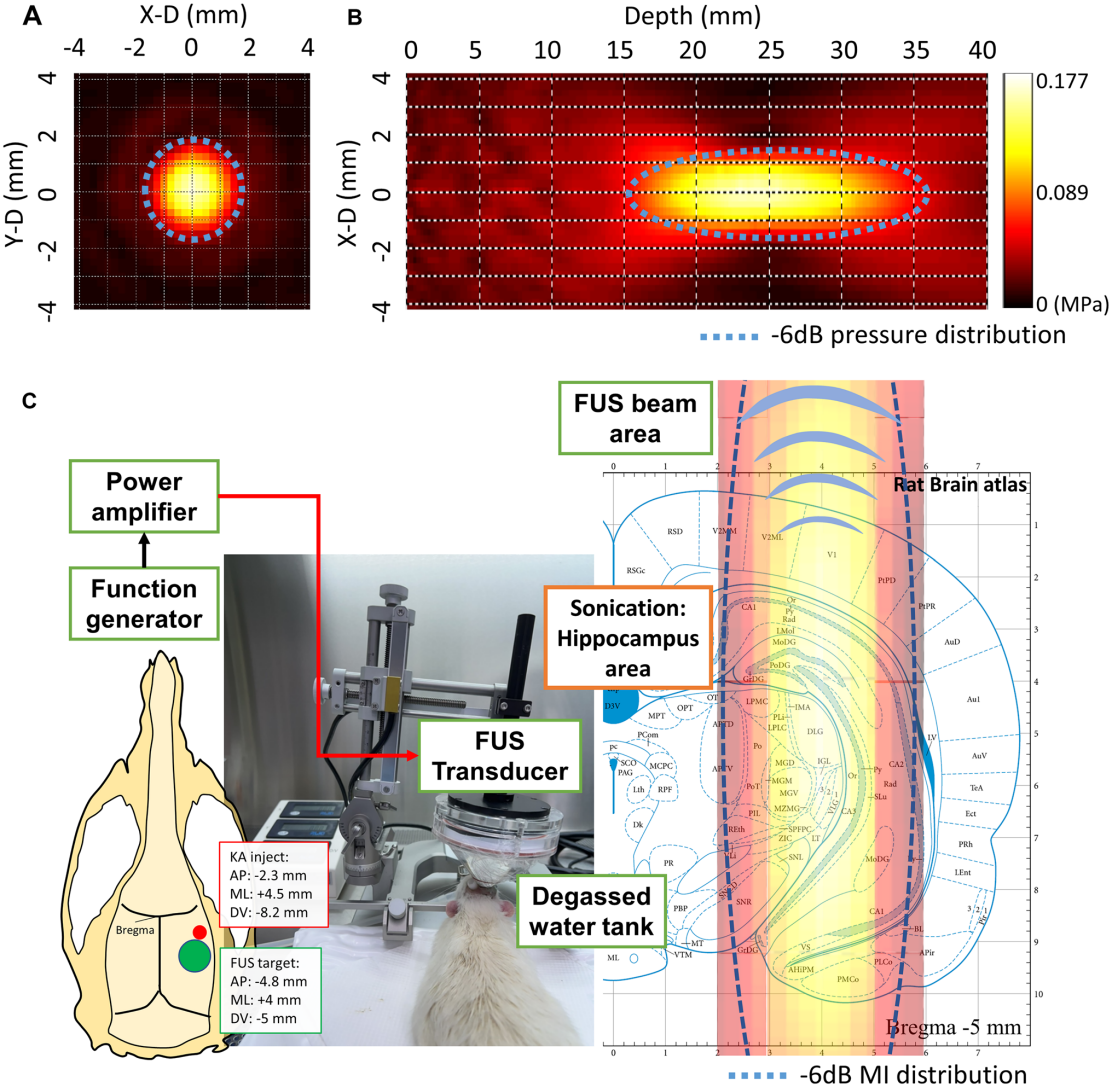
**A**, **B** Measured FUS distributions along the cross-sectional and axial planes. **C** Experimental setup showing the KA injection site (amygdala) and atlas overlay with the FUS beam, indicates that FUS energy covers the hippocampus area (marked as CA1, CA2, and CA3 in the atlas [20]). Dashed contours outline the − 6 dB pressure distributions

A focused ultrasound transducer (Sonic Concept, U.S; fundamental frequency = 0.5 MHz, radius curvature = 64.3 mm) transmitted energy to the skull and tissues by a custom-made coupler containing water. Burst-tone signals with carrier frequency of 0.5 MHz were triggered by a function generator (A33420, Agilent, USA), with the radio frequency signals amplified by a radiofrequency power amplifier (240L, E&I, USA). The acoustic pressure was measured by a calibrated needle-type hydrophone (Reson, TC 4038, Goleta, California, USA) under the environment in a deionized/degassed water filled container with the ultrasound path inserted by rat skull bones (*n* = 3) to estimate the averaged transcranial pressure. The diameter and length of the − 6 dB dimension of pressure field were 4 mm and 23 mm, respectively, aimed at the right hippocampus (Fig. 1A, B). Based on our prior experience [16], the pulsed FUS transcranial exposure level was set to a peak negative pressure of 0.177 MPa (considering 23% transcranially attenuated acoustic pressure with an ex vivo rat skull placed between the transducer and hydrophone), which was equivalent to a level of 0.25-MI and spatial-peak pulse-average intensity (I_SPPA_) 1.67 W/cm^2^. The PRF of exposure was set to 100 Hz with the attempt to reduce audible effect and sensible differences [21, 22], and duty cycle was set to 30% based on our previous optimal setting [16]. These settings were equivalent to 0.5 W/cm^2^ of I_SPTA_, which is under the limitation of FDA guidelines on ultrasound imagers (i.e., 0.72 W/cm^2^ I_SPTA_) and our settings did not produce thermal effect in our previous study [16]. Exposure duration was for three consecutive 10-min “on” periods segmented by 5-min intermittent “off” periods. After each sonication, ultrasonic gel was reapplied to secure ultrasound coupling.

### FUS Sonication Protocol

**Fig. 2:**
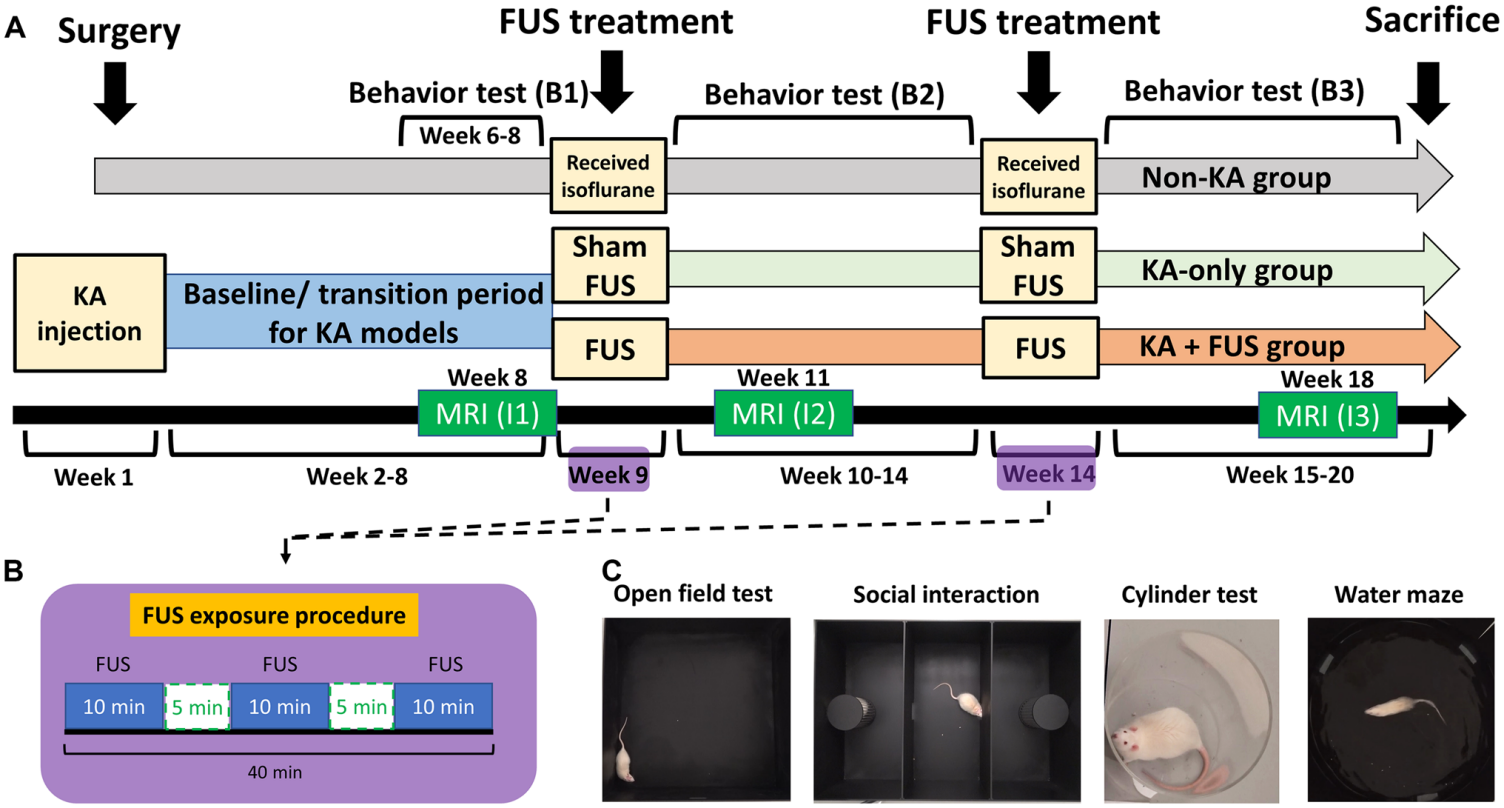
**A** Time course of the overall experimental design. **B** FUS sonications paradigm design. **C** Behavioral examination design

Figure 2A illustrates the FUS sonication protocol and time course. In week 1, KA was injected into the right amygdala. During weeks 6–8, behavioral tests were administered weekly to serve as the baseline (denoted as B1). At week 9, FUS stimulation was delivered at 0.25-MI, 100 Hz PRF, 30% duty cycle, I_SPTA_ 0.5 W/cm^2^, and three consecutive 10-min pulse trains with 5-min intermittent segmentations (Fig. 2B). Behavioral tasks were repeated at weeks 10–14 (denoted as B2). At week 14, animals participated in the 2nd FUS stimulation session, followed with behavioral testing during weeks 15–20 (denoted as B3). Behavioral tasks (detailed below) comprised an open-field exploration test, social interaction assay, cylinder limb motor test, and a water maze learning test (Fig. 2C). A T2-weighted MRI was obtained at week 8 (pre-FUS, denoted as I1), week 11 (post-first FUS, denoted as I2), and week 16 (post-second FUS, denoted as I3).

During FUS exposure, isoflurane (2%) was used to mildly anesthetize and stabilize the animals during sonication. To exclude confounding effects from anesthesia, a non-KA animal group (*n* = 15) was given only isoflurane and another KA-only animal group (*n* = 8) received isoflurane inhalation and sham FUS exposures (i.e., 0 MI and I_SPTA_ 0 W/cm^2^). Handling, ultrasound gel, and treatment otherwise were the same as that used for the groups exposed to FUS (KA + FUS group, *n* = 6).

### Magnetic Resonance Imaging

Neuronal damage caused by KA injection was evaluated with a small animal 7 T MRI (Bruker BiosSpec 30/70). Turbo-spin-echo T2-weighted images were acquired at three time points to longitudinally evaluate any anatomical changes due to KA or FUS. MRI parameters were TR/TE = 2500/33 ms; FOV = 25 × 25 mm^2^; in-plane resolution = 0.1 × 0.1 mm^2^; and slice thickness = 1 mm. Volume changes of hippocampus and striatum were quantified relative to the contralateral untreated sites. Hippocampus and striatum volumes were defined by marking the voxels from each image slide and then summed through the entire slides by a blinded rater (via the Area Measure function of Image J).

### Behavioral Tasks and Analysis

#### Open-Field Test

This task was performed to assess animal anxiety. A box (100 × 100 × 50 cm) made of plastic comprised a perimeter area (3/4 of the total area) and a center area. The percentages of time spent and distances traveled in each area were measured over a 5-min period by video monitoring. Amount of time spent near the “protected” walls versus center open space is taken as a surrogate measure of anxiety [12].

#### Sociability Test

Testing was in a rectangular (122 × 80 × 40 cm) white plastic box with three compartments, i.e., left, middle, and right, each one-third of the total area, with holes for moving between compartments. Two wire mesh cages (diameter 12 cm; height 25 cm) were placed in the left and right compartments facing the middle compartment. The subject rat was placed in the middle of the box and allowed to explore and habituate for 10 min. An age-matched male SD rat unfamiliar to the test rat was placed in the wire cage of the left compartment, and then the subject rat was placed again in the middle of the box. Over the subsequent 10 min, the time the test rat spent exploring each wire cage was measured [23].

#### Cylinder Test

This task evaluates motor function and limb usage. The subject rat was placed into a clear acrylic cylinder (height, 50 cm; diameter, 23 cm) for 5 min with motor activity observed by video monitoring. Forelimb usage was measured by counting the left and right paw wall contacts and by observing rearing in the form of standing on the hind paws with or without support from the cylinder wall. The forelimb usage was calculated as the percentage of all forelimb actions including left paw touching only, right paw touching only and double paw supporting. Only touching actions from the left or right paw are presented [24].

#### Morris Water Maze Test

This test assesses memory and learning in a Morris water maze with a black circular pool (120-cm diameter, 100 cm high with black wall) filled with water (30 cm depth) and virtually divided into four equivalent quadrants. A 2-cm submerged platform (10-cm diameter) was placed in the center of the pool. A trial began by placing the rat into the water facing the wall of the pool. The rat then was guided to the platform within 60 s and allowed to stay on the platform for 10 s. The trial is repeated 8 times starting from different quadrants. The time to swim to the invisible platform and time spent in the platform area is taken as a measure of learning and memory. Whishaw’s error index calculated the efficiency of a chosen path to approach the platform [25].

Behavioral data was recorded and analyzed by a blinder rater with video-tracking systems from Swart V3.0 (Panlab, Barcelona, Spain). A minimum of three animals from each of the non-KA, KA-only and KA + FUS group in each behavioral task were studied.

### Immunohistochemistry Examinations

We previously found hippocampal shrinkage and inflammation in KA-epilepsy models, as shown with hematoxylin and eosin and GFAP stains, respectively [16]. Both abnormalities improved with sonication using 0.25-MI and I_SPTA_ 0.5 W/cm^2^ parameters without thermal damage (Supplementary S2) [16].

Based on the neuroprotective hypothesis that might link to FUS-assisted anti-inflammatory effect [14, 16], we further examined the expressions of the ionized calcium-binding adaptor molecule 1 (IBA-1) and TNF-α [26, 27] after two FUS treatments. Non-KA, KA only, and KA + FUS animals were anesthetized with isoflurane and sacrificed by transcardial perfusion with 0.9% saline at week 20. Brains were removed and fixed in 10% buffered neutral formalin. Fixed brains were dehydrated by gradient osmosis in 10%, 20%, and 30% sucrose solutions at 4℃. After dehydration, the brains were cut into a series of coronal blocks and embedded in optimal cutting temperature compound and were serially sectioned at 20 μm for IBA-1 (GeneTex, GTX635399, 1:500) and TNF-α (GeneTex, GTX110520, 1:500) of IHC staining. Histological changes in hippocampus and striatum were evaluated after two I_SPTA_ 0.5 W/cm^2^ FUS treatments. The relative change of positive signals in IHC staining was quantified while using the contralateral untreated site as the baseline (via Image J [25]).

### Statistical Analysis

Statistical analysis was performed using R Statistical Software (v. 3.6.3) and RStudio (v. 1.2.5042). MRI, behavioral, and histology data were analyzed using one-way ANOVA with REGWQ test post hoc analysis at an alpha criterion of 0.05, with comparison of the KA + FUS group to the non-KA group and KA-only group. For sociability test, two-tailed/paired Student’s *t*-tests at an alpha criterion of 0.05 were used for comparison of the habitation and stranger rat interaction of the aforementioned three groups.

## Results

### MRI Analysis

**Fig. 3:**
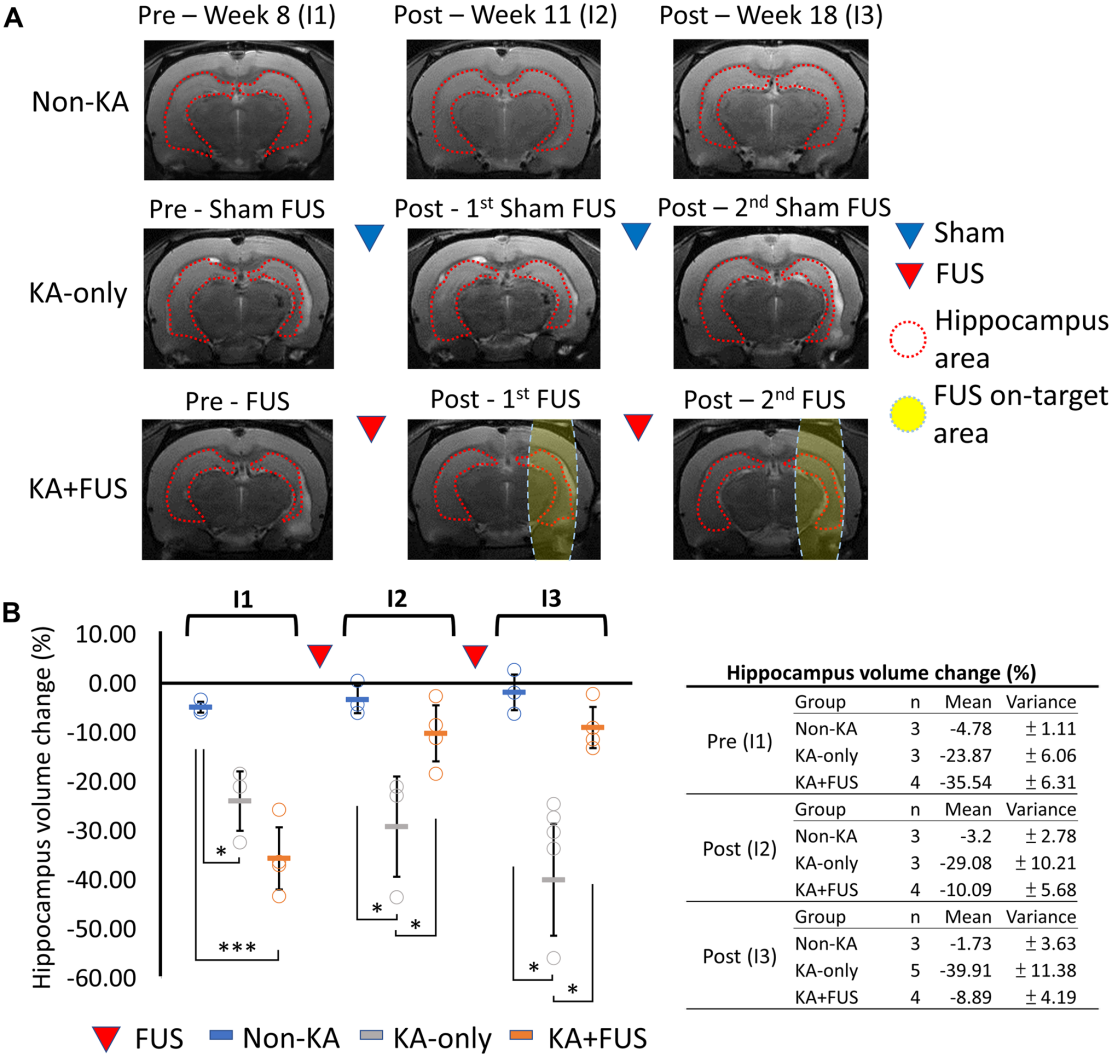
MRI analysis for various group after each course of sham-FUS or active FUS. **A** Typical case of the hippocampal volume according to the MRI among testing groups. **B** The MRI scanned at pre-FUS, post-first FUS, and post-second FUS are denoted as I1, I2, and I3, respectively, with the numerical analysis of the MRI analysis summarized in the table. *p < 0.05; ***p < 0.001

Hippocampal volume changes for non-KA, KA-only, and KA + FUS groups are shown in Fig. 3. Non-KA animals presented symmetric hippocampal volumes between hemispheres throughout the observation period. Apparent structural changes could be observed in the KA-only and KA + FUS groups prior to the first FUS treatment (week 9, Fig. 3A). Right hippocampal volumes significantly declined after right amygdala KA injection (group effect, *F* (2, 9) = 20.85, *p* < 0.01; − 23.87 ± 6.06% in KA-only animal and − 35.54 ± 6.31% in KA + FUS animal compared to the contralateral hippocampus; *p* < 0.05 by post hoc analysis), confirming the neuronal toxicity of KA. Hippocampal volume reductions were partially reversed after the first and second FUS treatments (− 10.09 ± 5.68% and − 8.89 ± 4.19%, respectively). These volumes differed significantly from the volumes in the KA-only group at weeks 15–20 (*p* < 0.01; Fig. 3B). No hippocampal volume restoration was observed in the KA-only group at weeks 10–20 after two sham procedures.

**Fig. 4:**
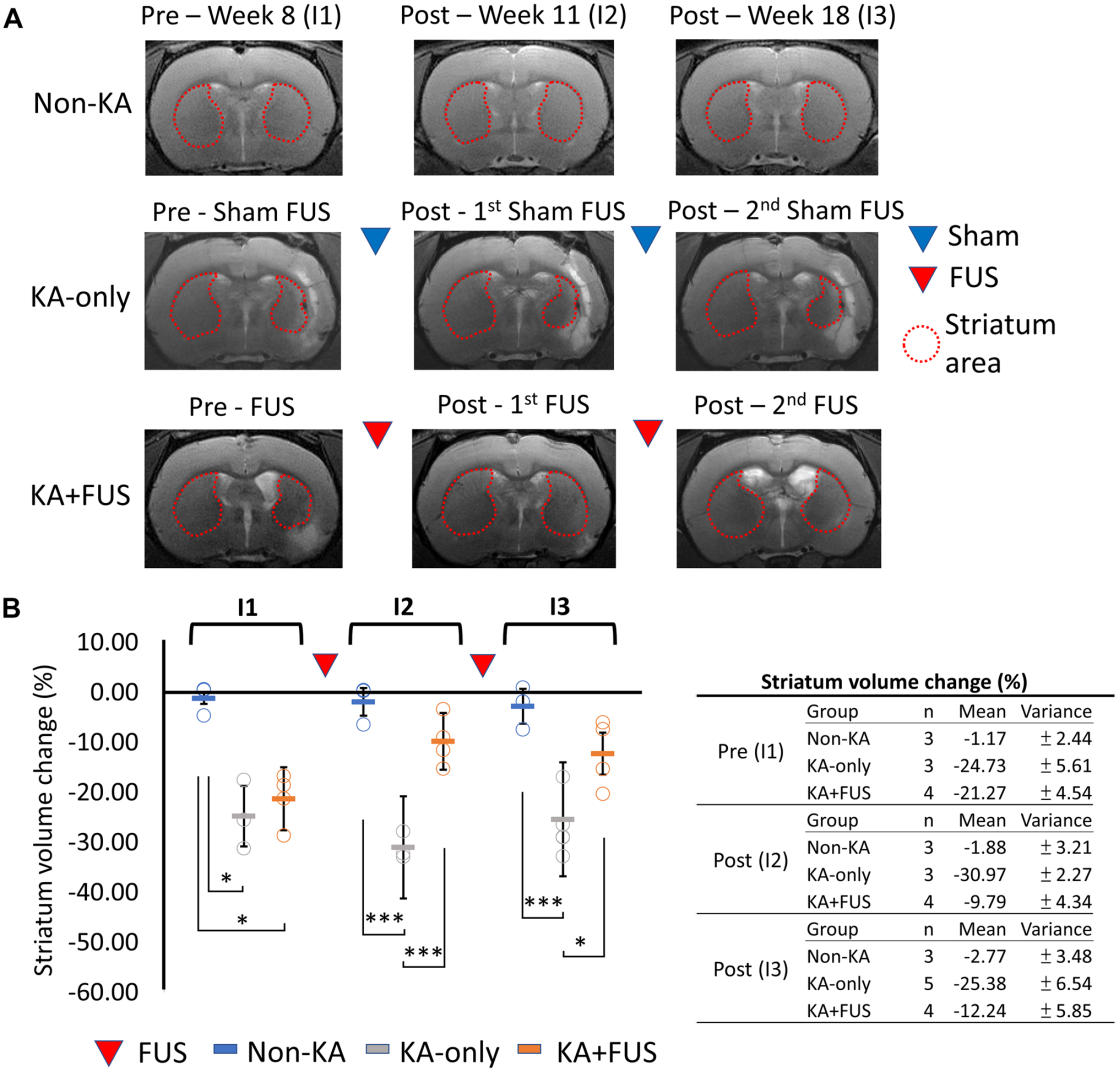
MRI analysis for various group after each course of sham-FUS or active FUS.** A** Typical case of the striatum volume changes in MRIs among the testing groups. **B** The MRI scanned at pre-FUS, post-first FUS, and post-second FUS are denoted as I1, I2, and I3, respectively, with the numerical analysis of the MRI analysis summarized in the table. *p < 0.05; ***p < 0.001

Changes in striatal volume among non-KA, KA-only, and KA + FUS groups were also measured, as shown in Fig. 4. Non-KA animals presented a symmetric striatal volume between hemispheres (Fig. 4A). ANOVA indicated that the striatal volume differed among the three groups (group effect, *F* (2, 9) = 17.94, *p* < 0.01). KA injection significantly reduced striatal volume (− 24.73 ± 5.61% in KA-only animals and − 21.27 ± 4.54% in KA + FUS animals compared to the contralateral striatum; *p* < 0.01 by post hoc analysis). After two FUS sessions for the KA + FUS group, the striatal volume loss ipsilateral to the KA-injection showed noticeable recovery (improved to − 9.49 ± 4.34% and − 12.24 ± 5.85% of baseline levels, significantly better than the volumes in the KA-only group at I1 and I2 time points, *p* < 0.05 (Fig. 4B)). In contrast, in the KA-only group, striatal volume did not recover throughout weeks 10–20, after sham sonications. The overall quantitative anatomical preservations of hippocampus and striatum in association with FUS pulsations can be compared from the degree of volume changes in the treatment groups via longitudinal MRI analysis shown in Figs. 3 and 4 (more detailed brain region segmentations to illustrate hippocampus/striatum volume changes are provided in Supplementary Fig. S3 and Supplementary Raw S1).

### Behavioral Analysis

#### Open-Field Test

**Fig. 5:**
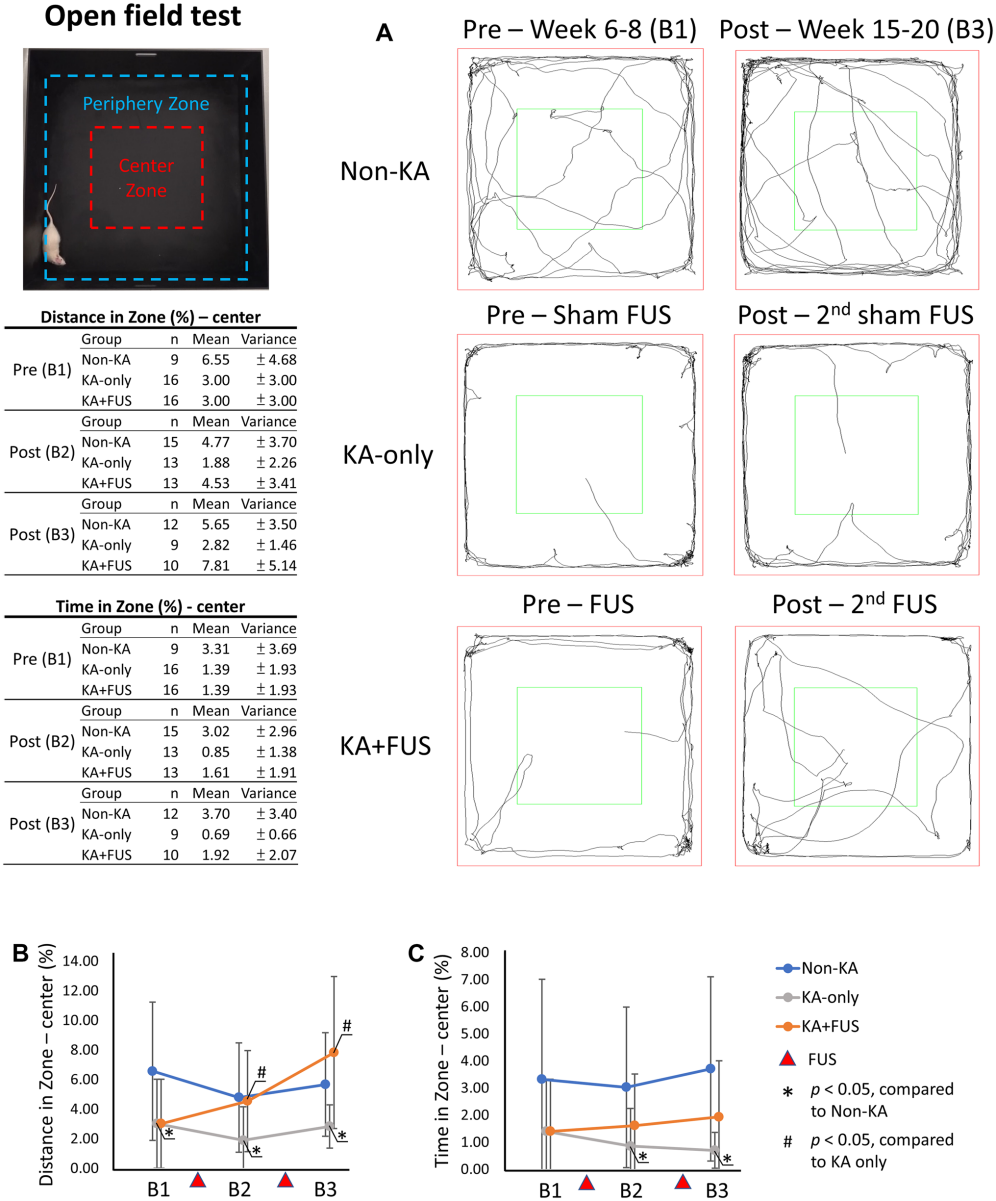
Summary of the anxiety analysis via open-field testing. **A** Typical trace diagrams of open-field testing among the three groups. **B**, **C** Comparison of the distance and time in zone among various groups (observation period: weeks 6–20). The behavioral tasks were tested at pre-FUS, post-first FUS, and post-second FUS time points, denoted as B1, B2, and B3, respectively

In the open-field task, KA-injected animals traveled shorter distances and resided for less time in the center area at weeks 6–8, reflecting less willingness to explore and presumably greater anxiety (Fig. 5A). The travel distances in the center area differed among the three groups (group effect, *F* (2, 38) = 3.56, *p* < 0.05) and lasted for the remainder of the experiment (all *p* < 0.05). Travel distances in the center area in the KA-only group decreased and significantly differed from the non-KA group (3 ± 3% vs. 6.55 ± 4.68%, post hoc analysis, *p* < 0.05) (Fig. 5B). The time in the center area did not differ among the three groups at weeks 6–8 (group effect, *F* (2, 38) = 2.1, *p* = 0.14) but differed for the remainder of the experiment (group effect, *F* (2, 38) = 3.63, *p* < 0.05). During weeks 6–8, the KA-only group spent less time in the center area (1.39 ± 1.93%) than did the non-KA (3.31 ± 3.69%) (Fig. 5C) animals. KA-only animals consistently traveled smaller distances (2.82 ± 1.46%; *p* < 0.05) and resided for less time in the center zone (0.69 ± 0.66%; *p* < 0.05) (see Fig. 5B, C).

In contrast, the distance traveled and time in the “less safe” center zone in the KA + FUS group both increased when compared with the KA-only group. After the 1st FUS, the distance traveled in the center zone of the KA + FUS group was 4.53 ± 3.41% at weeks 10–14 (*p* < 0.05 vs. 1.88 ± 2.26% of the KA-only group) (Fig. 5A). Two courses of FUS stimulations further increased exploratory performance up to 7.81 ± 5.14% at weeks 15–20 (*p* < 0.05 vs. KA-only group) and slightly increased the time in the center zone (no significant difference between the KA + FUS group and the non-KA group; see Fig. 5B). Open-field testing suggested that two courses of FUS pulsations increased exploration and reduced anxiety in the KA-lesioned animals.

#### Sociability Test

**Fig. 6:**
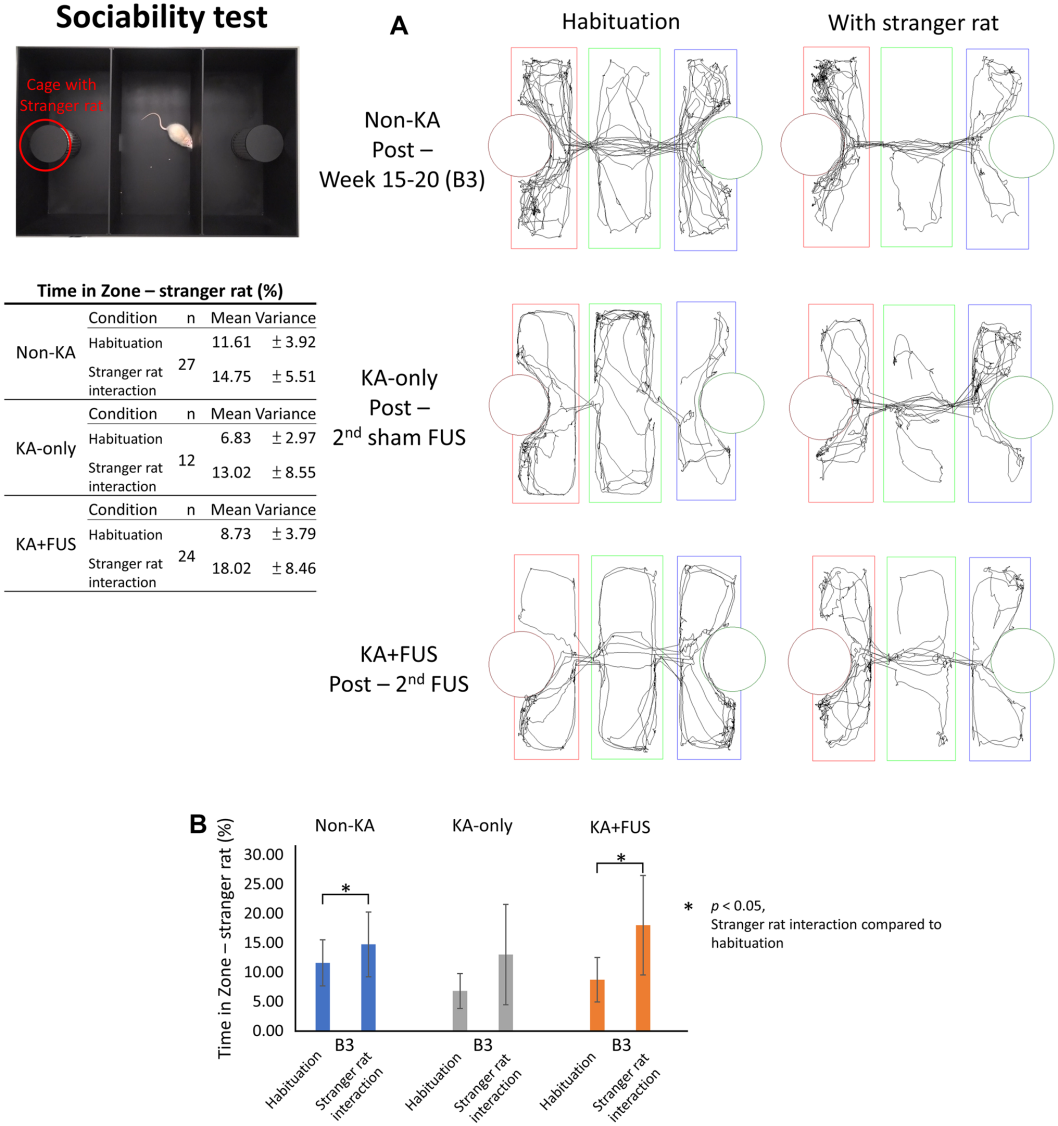
Summary of the sociability analysis via the sociability test environment setup. **A** Typical trace diagram for sociability test among the testing groups. **B** Analysis of time in zone (%) among various groups for the observation period weeks 15–20. The behavioral tasks tested at the 2nd FUS period are denoted as B3

In the sociability environmental setup, test animals from the non-KA group spent more time exploring the zone near the stranger rat (Fig. 6A), showing greater scalability, compared to time during the solo habituation period (14.75 ± 5.51% vs. 11.61 ± 3.92%; *p* < 0.05). When only analyzing the time change compared to the habituation period, the amount of time spent near the stranger was also significantly increased (18.02 ± 8.46% in zone of the stranger rat vs. 8.73 ± 3.79% during solo habituation; *p* < 0.05) in the KA + FUS group after two FUS treatments. When comparing to the KA-only animals, time increase in FUS + KA animals was noticeable. This improvement cannot be observed from the KA-only group (13.02 ± 8.55% vs. 6.83 ± 2.97%, respectively; no significantly difference; Fig. 6B). This indicates that the decline in sociability caused by KA can be improved after two-course FUS pulsations.

#### Cylinder Test

**Fig. 7:**
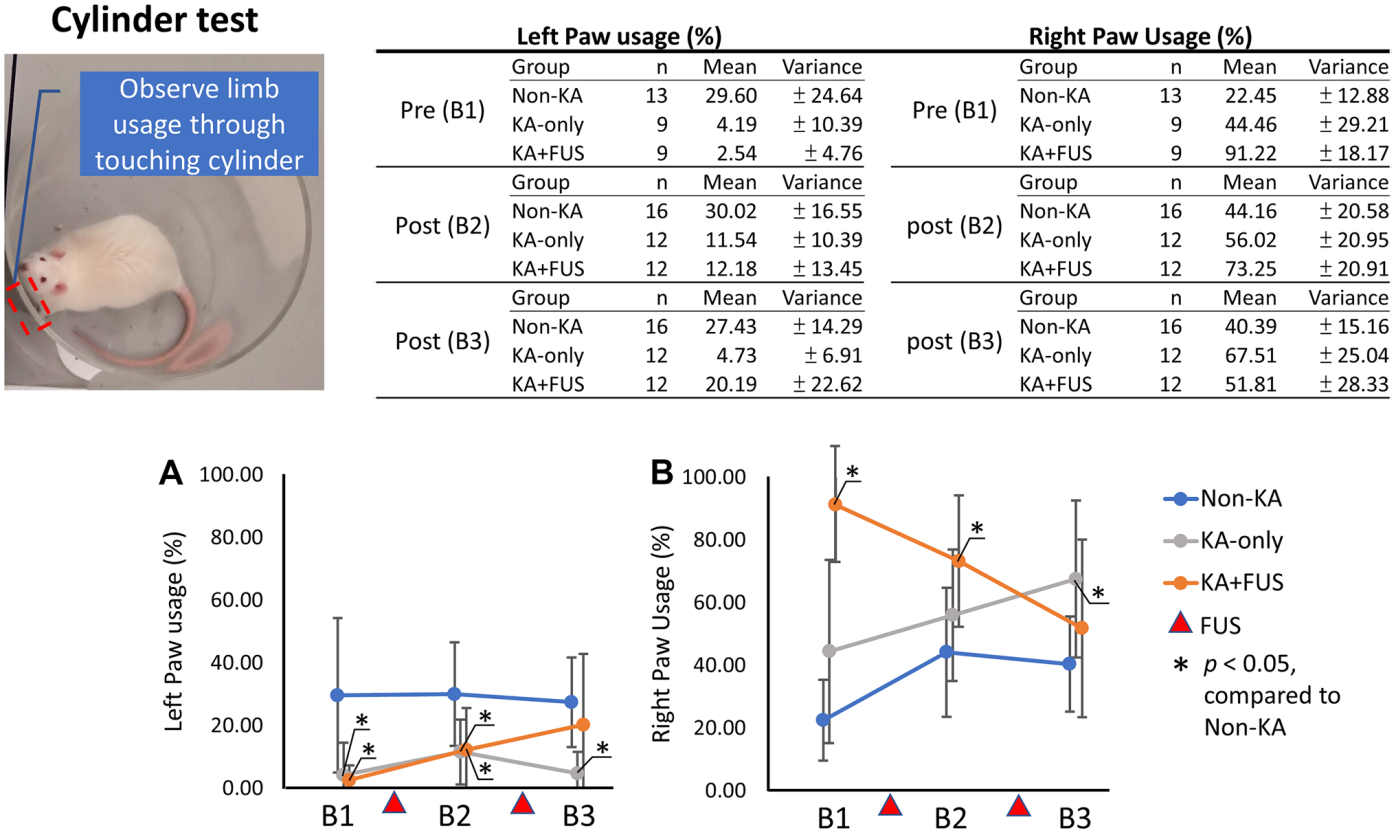
Summary of forelimb usage via cylinder test among various groups over the observation period weeks 6–20. **A** Left paw usage (in %). **B** Right paw usage (in %). The behavioral tasks were tested at pre-FUS, post-first FUS, and post-second FUS, and denoted as B1, B2, and B3, respectively

Symmetry of motor performance was evaluated by cylinder testing. Non-KA animals presented symmetric usage of the left and right paw after two sham FUS procedures, while KA animals obviously decreased their left paw usages (group effect, *F* (2, 28) = 8.04, *p* < 0.01). KA-only animals showed significant asymmetry of limb usage (*p* < 0.05 by post hoc analysis), with left (contralateral to KA injection) forelimb usage merely 4.19 ± 10.39% in the KA-only group and 2.54 ± 4.76% in the KA + FUS group before FUS stimulation (Fig. 7A), which were both significantly lower than right forelimb usages (group effect, *F* (2, 28) = 27.75, *p* < 0.001). Asymmetry of limb usage continued to increase by weeks 15–20. The right limb usage in the KA-only group increased from 56.02 ± 26.95% after the first sham treatment (*p* < 0.05) to 67.51 ± 25.04% after the second sham treatment (*p* < 0.05). In contrast, the KA + FUS group presented a progressive increase in left forelimb usage from 2.54 ± 4.76% after the 1st FUS pulsations at weeks 6–8 ( *p* < 0.05) to 20.19 ± 22.62% after two FUS treatments at weeks 15–20 (*p* < 0.05). Right forelimb usage in KA + FUS animals showed a trend toward decreased usage from 91.22 ± 18.17% before the first FUS treatment at weeks 6–8 to 51.81 ± 28.33% after the second FUS treatment at weeks 15–20 (Fig. 7). These findings suggest that two courses of 0.25-MI FUS pulsations largely restored the balanced limb usages in KA-lesioned animals.

#### Morris Water Maze Test

**Fig. 8:**
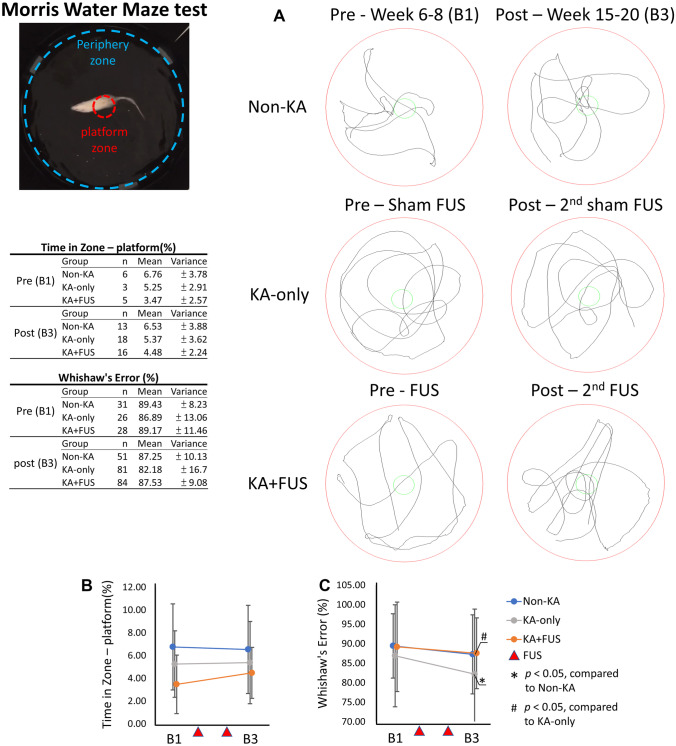
Morris Water Maze analysis for various group after two courses of sham-FUS or active FUS. **A** Comparison of the trace diagram for MWM test among the testing group. **B** Comparison of the time in zone and Whishaw’s error for various groups over the observation period weeks 6–20. The behavioral tasks were tested at pre-FUS and post-second FUS periods, denoted B1 and B3, respectively. The statistics were tabulated

Memory was evaluated by the Morris Water Maze test. During the early phase observation at weeks 6–8, non-KA animals showed trends toward increasing the time for learning among the three animal groups (6.53 ± 3.88%, compared to 5.25 ± 2.91% in KA-only animals and 3.47 ± 2.57% in KA + FUS animals; *F* (2, 11) = 1.13; *p* = 0.36; Fig. 8A, B). All groups showed similar Whishaw’s errors, which all exceeded 85% in the baseline period, implying that KA injection may not influence memory function until after weeks 6–8.

During the late phase observations at weeks 15–20, the KA-only group did not show increased time in the hidden platform zone, reflecting limited learning of platform location. The time in the hidden platform zone did not differ among the three groups (group effect, *F* (2, 44) = 1.31, *p* = 0.28). However, the Whishaw’s error index significantly decreased in KA-only groups (declining to 82.18 ± 16.7%, group effect, *F* (2, 213) = 4.28, *p* < 0.05 by post hoc analysis, Fig. 8C), and was identified to be significantly different from non-KA animals with change of Whishaw’s error (87.25 ± 10.13%). Notably, the Whishaw’s error in the KA + FUS group of was also significantly different from that of the KA-only group (87.53 ± 9.08%; *p* < 0.05 by post hoc analysis). The data indicates that KA injection impairs memory and learning in a water-maze test, and two courses of FUS pulsations partially restored memory functions in the KA-exposed animals.

### Histology Analysis

#### IBA-1 and TNF-α

**Fig. 9:**
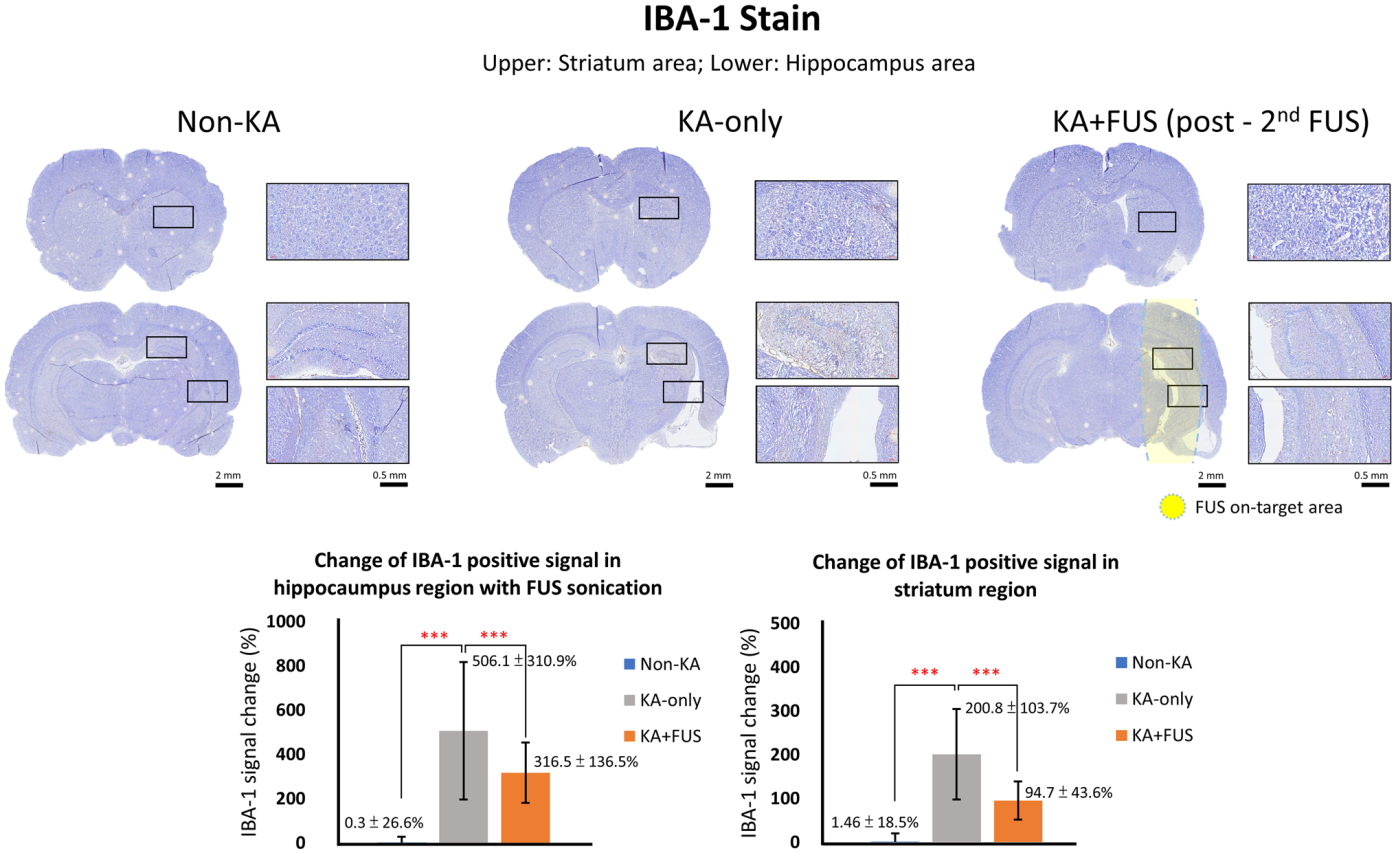
Comparison of the IBA-1 staining and IBA-1 positive signal change in hippocampal/striatal regions among the groups (the contralateral hippocampal/striatal region served as the basis). *p < 0.05; ***p < 0.001

To evaluate neuroprotective effect after two-courses of FUS pulsations, IBA-1 and TNF-α staining were conducted, with the non-KA/KA-only groups as comparisons. A slight decrease of right (KA-injection site) striatal region and apparent decrease of right hippocampal size could be observed in the KA-only group (Fig. 9). The marked microglia cells (i.e., IBA-1 signals) were increased in caudate and putamen (striatum), and CA1 and CA3 (hippocampus) regions in the KA-only group, indicating an inflammatory response after KA injection (group effect, *F* (2, 51) = 13.3, *p* < 0.001). IBA-1 signals were significantly detected in the KA-only group (506.1 ± 310.9% vs. 0.3 ± 26.6% in the non-KA group in hippocampal regions; *p* < 0.001; see Fig. 9).

In the KA + FUS group, animals showed apparently lower degrees of IBA-1 signals in FUS-treated hippocampus region than the signals in the KA-only group. IBA-1 signals were significantly suppressed in the KA + FUS group (316.5 ± 136.5% vs. 506.1 ± 310.9% in the KA-only group in hippocampal regions; *p* < 0.001; see Fig. 9). In striatum, IBA-1 expressions were also identified when comparing the KA-only and KA + FUS groups (group effect, *F* (2, 51) = 22.3, *p* < 0.001). IBA-1 signals were also identified to be significantly suppressed in the KA + FUS group, with the suppression effect in striatum similar to that in the hippocampus region (94.7 ± 43.6% vs. 200.8 ± 103.7% in the KA-only group; *p* < 0.001; see Fig. 9). This indicates that sonication decreased the inflammation effect of KA in hippocampus and also striatum, which suggests that the neuroprotective effect induced by FUS pulsations occurred in the off-target regions.

For TNF-α staining, none of the animal groups showed noticeable change even in the KA-only group. The addition of two courses of FUS pulsations produced neither gliosis nor a chronic inflammatory effect on the sonicated site when compared to the non-KA group, indicating that I_SPTA_ 0.5 W/cm^2^ FUS did not injure brain tissue with these parameters (Supplementary S4).

## Discussion

FUS neuromodulation has been considered a possible therapy for many clinical conditions [28, 29] and a modulator of sensory-evoked human cortical functions [30]. Epilepsy is one possible application and previous rodent studies of pentylenetetrazol- [9, 13] and kainic acid–induced seizures have demonstrated efficacy of FUS [12, 14, 16]. Clinical epilepsy typically presents with co-morbidities of depression, anxiety [31], low socialization [32], and behavior and cognition dysfunction (including memory loss) [33], all degrading the quality of life. In this study of the KA animal model of temporal lobe epilepsy, we utilized MRI to observe structural changes in hippocampus/striatum and four behavioral tests to measure cognitive function. A course of three sequential 10-min sonications were delivered to the right hippocampus at week 9 and week 14 after KA injection to the right amygdala. Sonication reduced anxiety, improved socialization, re-balanced asymmetrical limb usage, and partially restored memory and learning. The IHC staining demonstrated a neuroprotective effect with reduced inflammation accompanying FUS treatment. To our knowledge, these improvements have not previously been reported with animal models of epilepsy.

Previous literature has demonstrated a strong linkage between hippocampal injury and behavioral impairment. Intracerebral administration of KA induces neuronal loss in hippocampus, correlated with chronic behavioral dysfunction, including anxiety, impaired contralateral limb usage, lowered social interaction, and memory impairment [34, 35]. These impairments in the laboratory model parallel those occurring in patients with TLE, and so are useful for study of mechanisms and potential therapies [6]. MRI provides a noninvasive structural assay of the severity of KA toxicity, which can be correlated with behavioral testing. Hippocampus and surrounding regions are known to be involved in anxiety and memory formations and retrieval [36]. This correlation was again demonstrated in our study. In addition, the striatal shrinkage in KA models associates with movement disorders [37]. In the current study, FUS treatments partially restored use of the limb contralateral to injection, matching the reduced loss of striatal volume seen with MRI.

In FUS pulsations for epilepsy treatment, ideal parameters that correlate to efficacy remain obscure. Higher acoustic pressure and higher MI of FUS seemed to inhibit acute epileptic signals effectively [13]. However, higher MI with high duty cycle, causing high I_SPTA_, might cause transcranial heating lesions. A low MI but with a high duty cycle could also suppress EEG seizures and generate a neuroprotective effect [16], indicating that duty cycle is also a factor for parameter design. In a prior epilepsy treatment study, a neuroprotective effect was detected while PRF of duty cycle was set at 10 Hz and the neuroprotective effect was enhanced while increasing PRF (from 10 to 1000 Hz) [14]. These parameters were effective in other epilepsy treatment studies [9, 12, 15, 38], including use of continuous-wave sonication [39]. Duration and treatment frequency of FUS previously was found to improve KA-induced epileptic behavior for 3 weeks after 30-s FUS [12] and in our prior study, to suppress epileptiform EEG discharges for more than 1 month after 30-min FUS [16]. In the current study, 30-min FUS sonications were repeatedly applied at weeks 9 and 14 after KA injections. The first sonication was associated with decreased volume loss of hippocampus and striatum, which corresponded to improved behavior, while the second led to additional improvement lasting more than 2 months. KA control animals did not improve. An analog of anxiety measured in the open-field task also showed incremental improvement after the second sonication. These results suggest that the FUS ameliorating effect may outlast the exposure duration, and this long-term effect may depend on the total exposure duration and FUS treatment frequency.

FUS treatments have been demonstrated to provide neuroprotective effects in the KA-epilepsy model by demonstrating morphological and functional benefits [14]. An FUS-induced neuroprotective effect also can be supported by histological evidences including (1) previous study observing that FUS pulsations successfully suppressed epileptiform EEG patterns for 7 weeks and reduced inflammatory GFAP markers in hippocampus (Supplementary S2) [16]. (2) This current study shows significantly decreased levels of IBA-1 and microglial markers in hippocampus after two FUS treatments. Microglia activation has been reported to potentially play a neuroprotective role in brain [40], and might lead to reduced inflammation caused by kainic acid injection. (3) A previous study reported that FUS pulsations facilitated brain-derived neurotrophic factor secretions [41] and contributed to neuroprotective and neurogenesis effects in a vascular dementia animal model [42, 43].

We observed that neuroprotective effects and tissue restoration effects occurred not only at the hippocampal target sonication site but also outside the sonication zone in the striatum. This “widespread” FUS effect has been previously reported in studies to alleviate anxiety and depression in animal models produced by activating infralimbic cortex, with concurrent modulation of distant striatum and hippocampus [44]. A prior study attempted to observe this “widespread” effect via EEG to interpret this effect from brain-region inter-connected point of view, revealing that the FUS first activate the “on-target” EEG signals and then propagated to the surrounding “off-target” brain regions [45]. A similar role of networks was documented in a study of FUS-triggered enhancement of resting-state functional connectivity between the striatum and multiple cortical regions in primate brain network [46, 47]. In our current study, the neuropathological lesions induced by KA were found in hippocampus and striatum, and FUS pulsations targeting hippocampus also restored striatum functionality and morphology. This observation supports the existence of aberrantly “hyper-connected” functional or morphological connectivity [48], and documents a neuroprotective effect for both “on-target” and “off-target” brain regions in a network.

Sonication could produce changes due to nonspecific effects, such as vibratory or auditory actions [22] via indirect activation of auditory pathways rather than direct sonicated neuromodulation [49]. This confounding effect would be particularly apparent with ultrasound pulsations having PRF > 500 Hz [49]. In addition, a smooth rectangular envelope of pulsations minimizes the auditory confounding effect [50]. In our study, FUS pulsation with low PRF (100 Hz) and low exposure level (0.25-MI) was employed to reduce this confounding effect based on observations provided from literature [50, 51]. Although no control experiments were performed for this potential confounder, we did analyze the contralateral brain of the KA-only and KA + FUS animal as a partial control (Supplementary S5). No apparent volume change or IBA-1 expressions was identified in the contralateral hippocampus/striatum. This may imply that the auditory confounding effect plays an insignificant role in this study.

Interpretation of our findings may consider several additional limitations. We tested one set of sonication parameters, but have no way to know whether these are optimal. Our prior study indicated that there are levels above which tissue injury becomes evident and these are to be avoided in a neuromodulation protocol. Energy delivered by our optimal sonication parameters would be very different when traversing the thicker human skull, so parameter adjustments would be necessary for clinical use. We directed energy at the hippocampus but cannot rule out effects on neighboring structures, such as the entorhinal cortex, amygdala, or piriform cortex. Results seen with animal models of epilepsy do not always carry over to clinical practice, even though animal models are used extensively to screen anti-seizure medications. We have so far studied effects on volumetric MRIs, tissue histology, EEG spikes, bursts, and electrographic seizures, but not on behavioral seizures. Whether our observed beneficial anatomical and behavioral effects resulted directly from FUS or indirectly from reduction of seizures or from some other factors is unclear. In addition, our study time course was chronic for a rodent, but not long for a human. It is challenging to observe animals in the KA model for more than about 5 months. It was difficult simultaneously to monitor EEG and MRI, since our EEG electrodes induced strong susceptibility artifacts in the MR environment. Therefore, the EEG and behavioral seizure were not analyzed in animals receiving an MRI but instead from animals extracted from our previous study [16]. Our non-KA group also presented slight declines of right hippocampal volumes, possibly due to surgical injuries with sham injection in these rodent strains. Despite these limitations, this study suggests that two courses of three trains of FUS neuromodulation can protect hippocampal tissue and partially ameliorate behavioral deficits caused by kainic acid–induced seizures and tissue injury in the rat model. This furthers hope that modulatory FUS might be developed as a useful adjuvant treatment of clinical epilepsy.

## Conclusion

This study shows that two courses 5 weeks apart of three sequential same-day FUS treatments improved kainic acid–induced hippocampal and striatal volume loss as seen with sequential MRIs. FUS led to behavioral improvements related to increased exploration (a surrogate for decreased anxiety in a rat), and increased sociability and better use of the limb contralateral to KA injection. Learning to swim to a hidden platform declined with KA injections but subsequently improved after two FUS treatments. For all of these measures, two treatments were more effective than was a single treatment. Results in animal models of epilepsy do not always translate to clinical benefit, but this study provides hope that FUS neuromodulation might be developed as a useful adjunctive clinical epilepsy treatment.

## Supplementary Information

Below is the link to the electronic supplementary material.

**Supp. S1.** Typical EEG spikes, bursts and electrographic seizures in an animal receiving KA versus a control non-KA animal [16]. (TIF 1112 kb)

**Supp. S2.** Hematoxylin-Eosin (HE) and Glial Fibrillary Astrocytic Protein (GFAP) stains of the hippocampal region for the three tests groups in our previous study after one course of FUS pulsations [16]. (TIF 3020 kb)

**Supp. S3.** Longitudinal MRI analysis of hippocampus and striatum volume changes among various groups. The hippocampus and striatum regions were marked as red and green, respectively. (TIF 17069 kb)

**Supp. S4.** Comparison of the TNF-α staining among the testing group. (TIF 5821 kb)

**Supp. S5.** Comparison of the volume of MRI and IBA-1 positive signal in the contralateral (non-FUS) hippocampal/striatal regions among the testing group, no significant difference was found between groups. (TIF 478 kb)

**Supp. Raw_S1.** MRI raw data among various groups. (7Z 5103 kb)

Supplementary file7 (PDF 446 kb)

Supplementary file8 (PDF 442 kb)

Supplementary file9 (PDF 433 kb)

Supplementary file10 (PDF 444 kb)

Supplementary file11 (PDF 435 kb)

Supplementary file12 (PDF 434 kb)

## Data Availability

The authors confirm that the data supporting the findings of this study are available within the article and its supplementary materials.
