# Supplementary figures and images for: Pulsed Focused Ultrasound Reduces Hippocampal Volume Loss and Improves Behavioral Performance in the Kainic Acid Rat Model of Epilepsy
Source: Neurotherapeutics. 2023 Mar 14;20(2):502–17. doi: 10.1007/s13311-023-01363-7 (PMC10121983; doi:10.1007/s13311-023-01363-7)

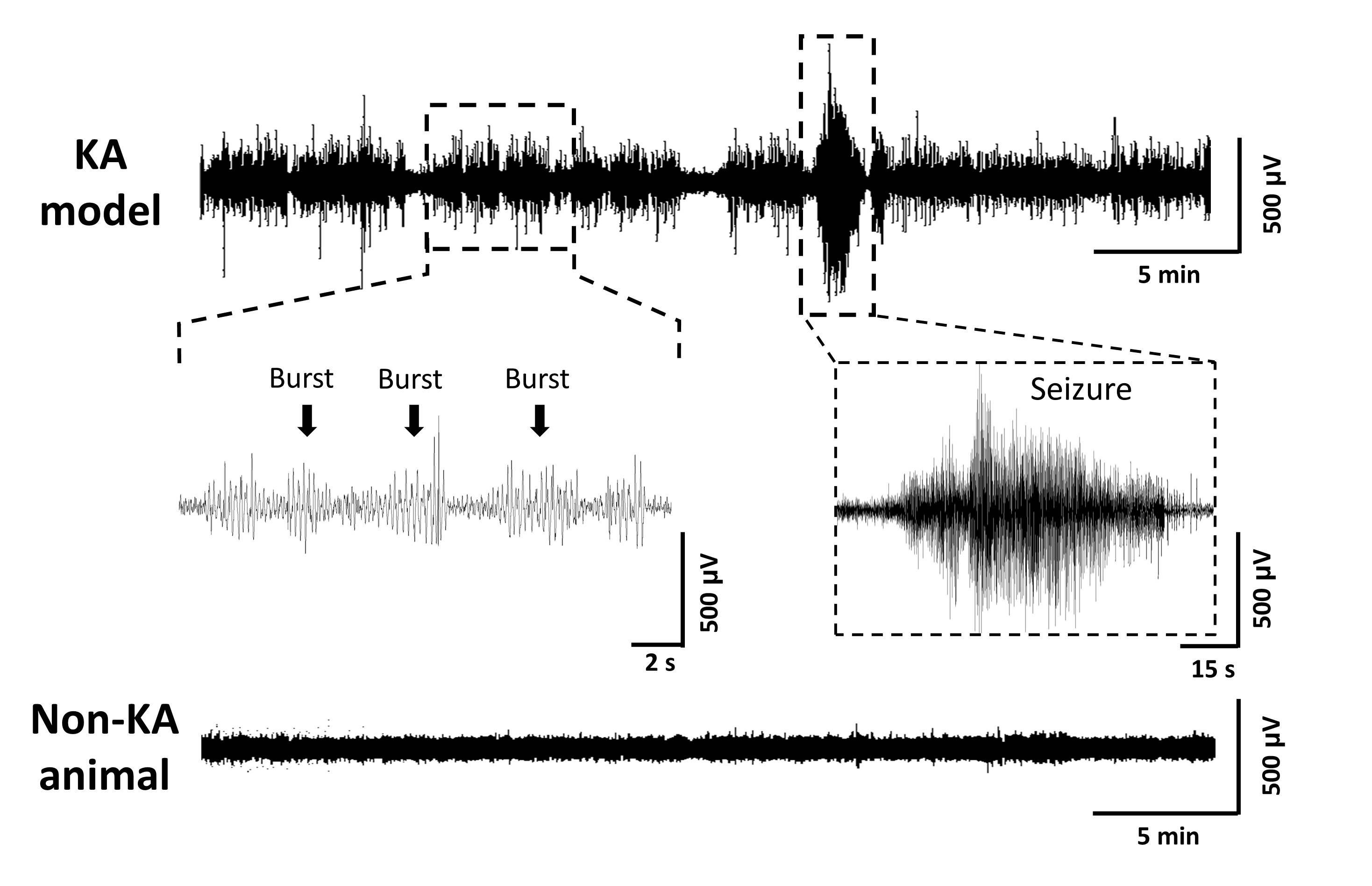

Supplement: Supplementary file 1 — Supp. S1. Typical EEG spikes, bursts and electrographic seizures in an animal receiving KA versus a control non-KA animal [16]. (TIF 1112 kb) [file 13311_2023_1363_MOESM1_ESM.tif]

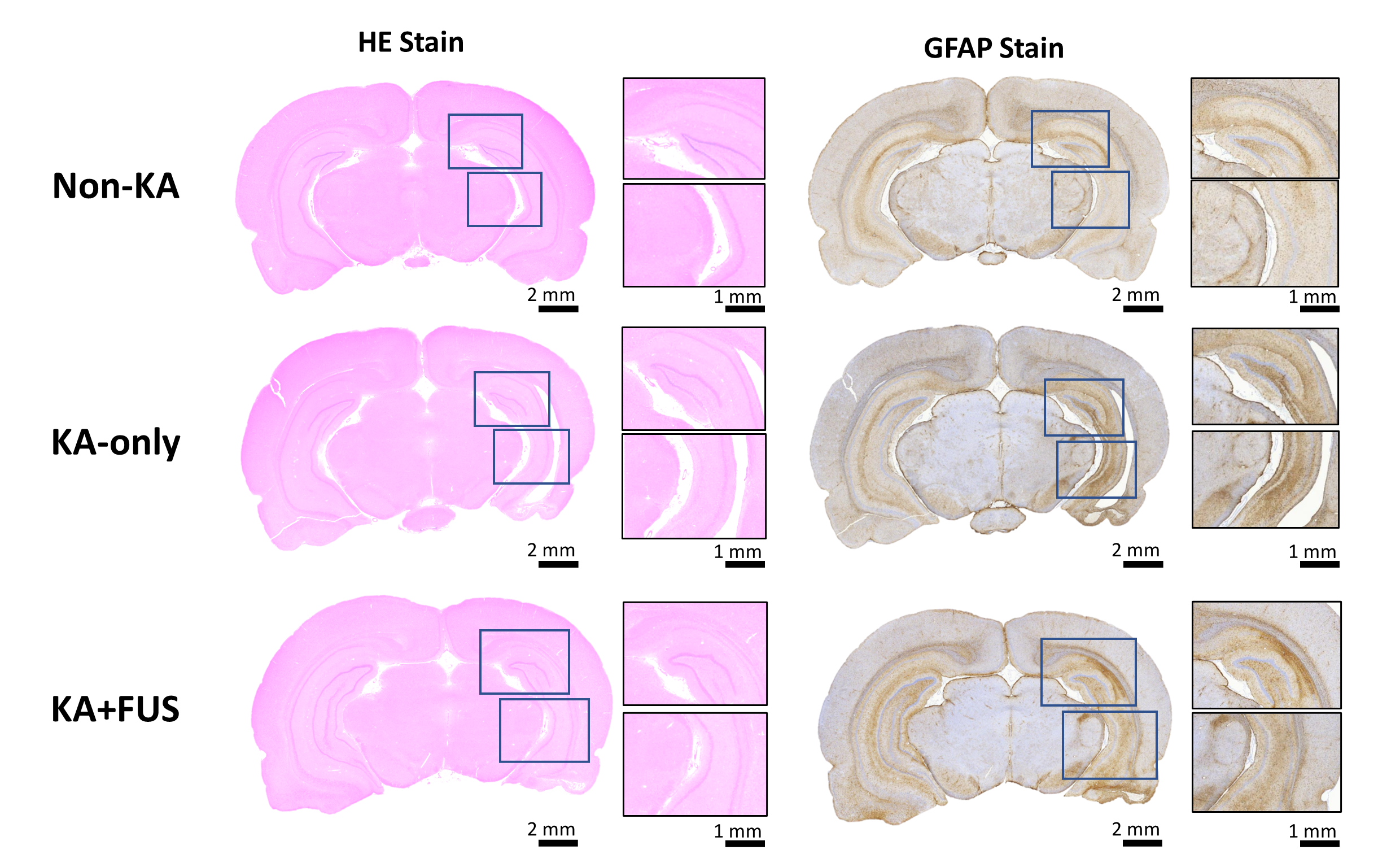

Supplement: Supplementary file 2 — Supp. S2. Hematoxylin-Eosin (HE) and Glial Fibrillary Astrocytic Protein (GFAP) stains of the hippocampal region for the three tests groups in our previous study after one course of FUS pulsations [16]. (TIF 3020 kb) [file 13311_2023_1363_MOESM2_ESM.tif]

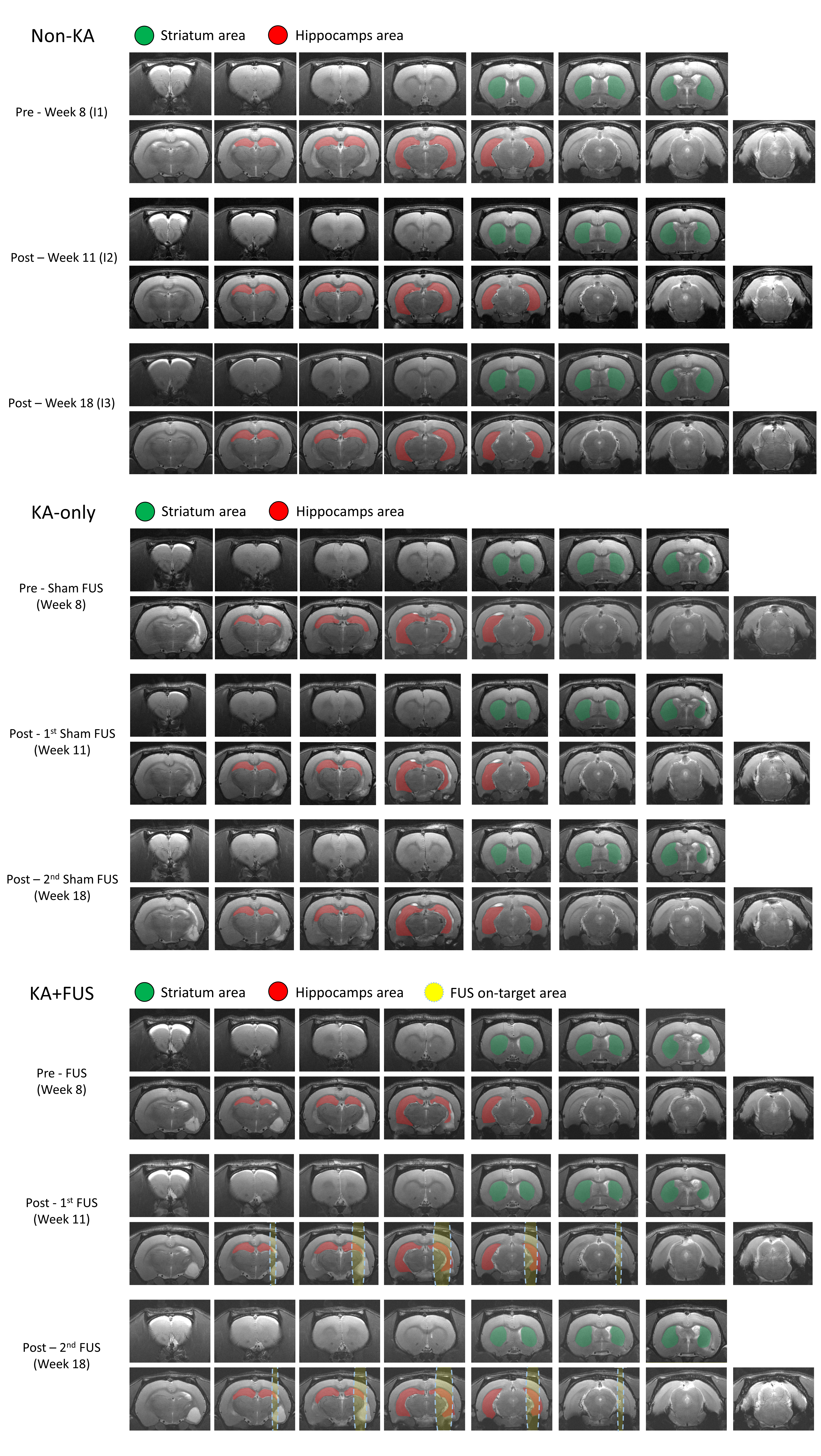

Supplement: Supplementary file 3 — Supp. S3. Longitudinal MRI analysis of hippocampus and striatum volume changes among various groups. The hippocampus and striatum regions were marked as red and green, respectively. (TIF 17069 kb) [file 13311_2023_1363_MOESM3_ESM.tif]

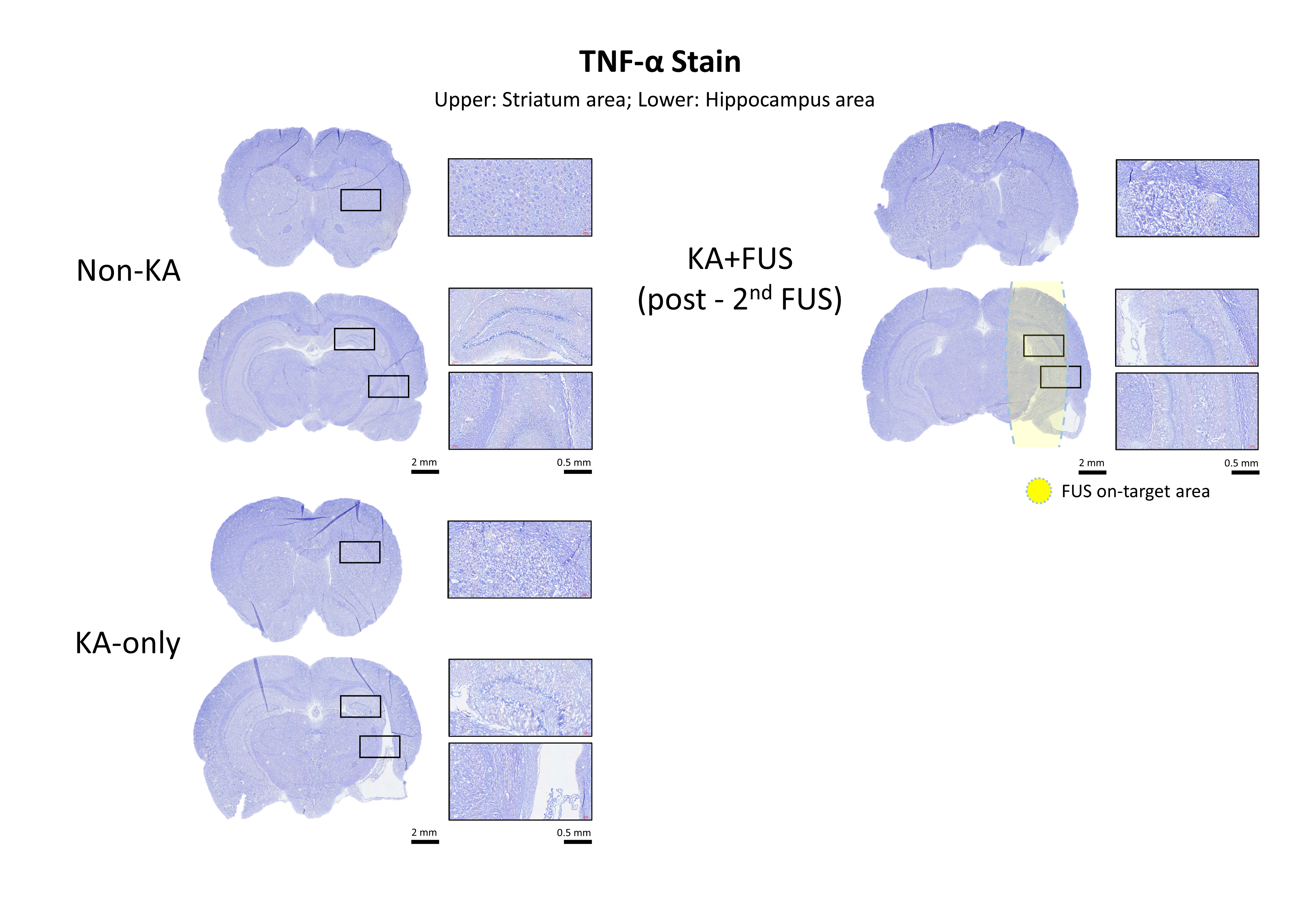

Supplement: Supplementary file 4 — Supp. S4. Comparison of the TNF-α staining among the testing group. (TIF 5821 kb) [file 13311_2023_1363_MOESM4_ESM.tif]

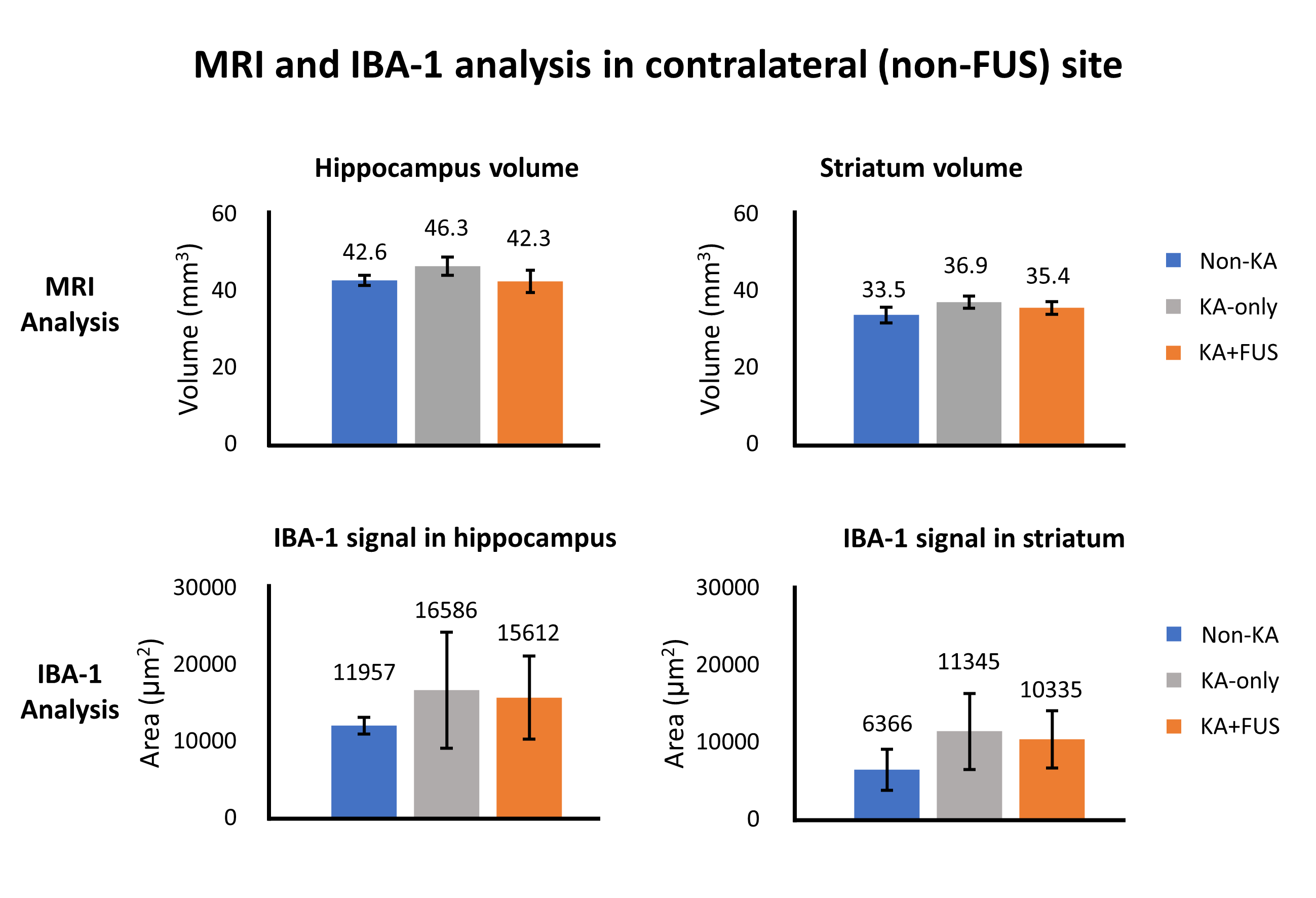

Supplement: Supplementary file 5 — Supp. S5. Comparison of the volume of MRI and IBA-1 positive signal in the contralateral (non-FUS) hippocampal/striatal regions among the testing group, no significant difference was found between groups. (TIF 478 kb) [file 13311_2023_1363_MOESM5_ESM.tif]
